# Supplementary material for: Oxidative Stress-Induced TRPV2 Expression Increase Is Involved in Diabetic Cataracts and Apoptosis of Lens Epithelial Cells in a High-Glucose Environment
Source: Cells. 2022 Apr 2;11(7):1196. doi: 10.3390/cells11071196 (PMC8998065; doi:10.3390/cells11071196)
Supplement: Supplementary file 1 [file cells-11-01196-s001.zip › cells-1600352-supplementary.pdf]

Supplementary Figures

Supplemental Methods

$[Ca^{2+}]_i$ measurement

HLEpiCs were incubated with Fura-2/AM (2 μM, Abcam, ab120873) and 0.02% Pluronic F-127 (Sigma-Aldrich, P2443-250G) in an incubator kept in the dark for 30 min at 37 °C. Basal intracellular Ca^{2+} concentration were measured for 10 min in a normal physiological saline solution containing (in mM) 140 NaCl, 5 KCl, 1 $MgCl_2$, 10 glucose, 1 $CaCl_2$, and 5 HEPES (pH 7.4). Ca^{2+} fluorescence was recorded using fluorescence microscopy. Changes in the $[Ca^{2+}]_i$ were expressed as the ratio of 340/380.

HLEpiCs were incubated with 10 μM Fluo-8/AM in an incubator kept in the dark for 30 min at 37 °C. Cells were stimulated with 2-APB in a Ca^{2+}-free buffer saline solution (0 Ca^{2+}-BSS), containing (in mM) 140 NaCl, 5 KCl, 1 $MgCl_2$, 10 glucose, 0.2 EGTA, and 5 HEPES (pH 7.4). When Ca^{2+} influx reached equilibrium, 1 mM extracellular Ca^{2+} was applied for 10 min. Ca^{2+} fluorescence was recorded using fluorescence microscopy. Changes in the $[Ca^{2+}]_i$ were expressed as the ratio of fluorescence intensity before and after the 2-APB or Ca^{2+} application (F_1/F_0).

HLEpiCs were incubated with 10 μM Fluo-8/AM in an incubator kept in the dark for 30 min at 37 °C. Cells were treated with 10 μM CRAC channels inhibitor BTP2 for 10 min in a normal physiological saline solution containing (in mM) 140 NaCl, 5 KCl, 1 $MgCl_2$, 10 glucose, 1 $CaCl_2$, and 5 HEPES (pH 7.4). When Ca^{2+} influx reached equilibrium, 250 μM 2-APB was applied for 10 min. Ca^{2+} fluorescence was recorded using fluorescence microscopy. Changes in the $[Ca^{2+}]_i$ were expressed as the ratio of fluorescence intensity before and after the 2-APB or BTP2 application (F_1/F_0).

Mitochondrial membrane potential assay

After the treatment in different groups, HLEpiCs were loaded with fluorescent probe Rhodamine 123 (Beyotime, C2008S) diluted at a ratio of 1:1000 in serum-free medium and cultured at 37 °C with 5 % CO_2 for 30 min. Serum-free cell culture medium was used to wash excessive fluorescent dye. Finally, the fluorescence signal was detected by a fluorimeter.

Supplementary Figures

1. Changes of TRPV2-mediated intracellular Ca^{2+} concentration of lens epithelial cells

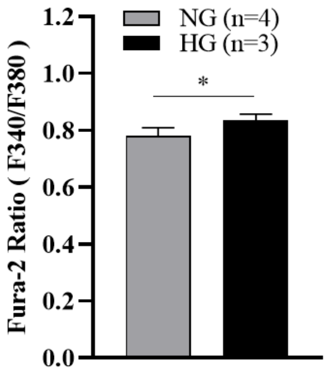

Figure S1. Summary data showing the Fura-2 fluorescence ratio (F340/F380) in HLEpiCs cultured in a normal-glucose (NG, 5.5 mM glucose and 20 mM mannitol) or high-glucose (HG, 25.6 mM glucose) medium for 7 days. Values are shown as the mean ± SEM; n = 3–4. **P* < 0.05 vs. NG analyzed by two-tailed unpaired Student's *t* test.

2. 2-APB-induced intracellular Ca^{2+} mobilization of lens epithelial cells

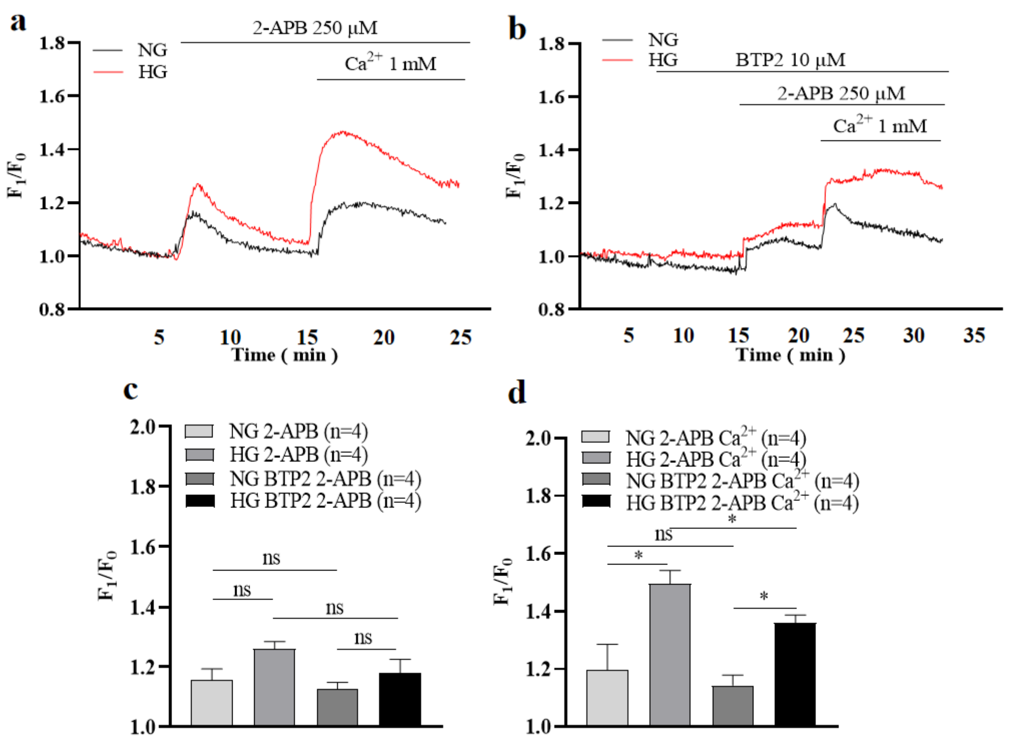

Figure S2. (**a-d**) Representative traces (**a-b**) and summary data (**c-d**) showing the changes in intracellular Ca^{2+} concentration of HLEpiCs cultured in NG or HG media for 7 days. (**a**–**b**,**c**) Intracellular Ca^{2+} release was activated by 2-APB (250 μM) and (**a**–**b**,**d**) Ca^{2+} influx was evoked by application of 1 mM extracellular Ca^{2+} in a Ca^{2+}-free buffer saline solution in HLEpiCs cultured in a normal-glucose (NG, 5.5 mM glucose and 20 mM mannitol) or high-glucose (HG, 25.6 mM glucose) medium for 7 days with or without BTP2 (10 μM, an inhibitor of SOCE) treatment. Values are shown as the mean ± SEM; n = 4. *P < 0.05 vs. NG 2-APB or NG 2-APB Ca^{2+} analyzed by two-tailed unpaired Student's *t* test; ns, not significant.

3. Role of TRPV2 in high glucose–induced apoptosis of lens epithelial cells

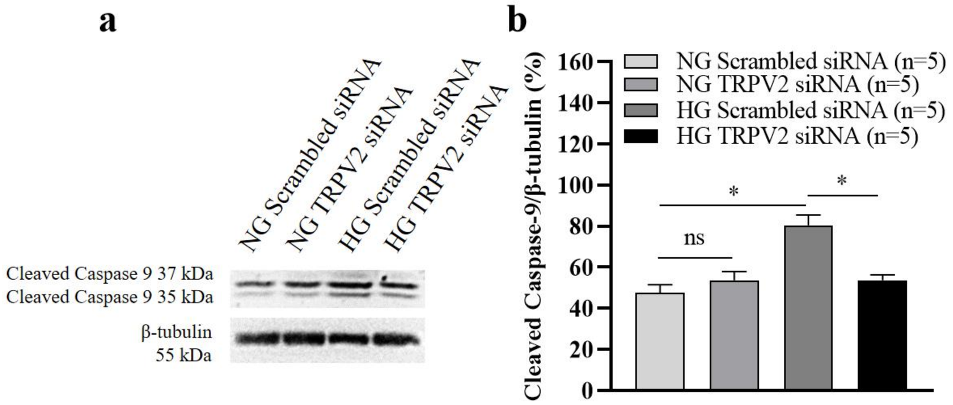

Figure S3. (a-b) Representative Western blotting images (**a**) and summary data (**b**) showing the levels of active caspase-9 in HLEpiCs cultured in a normal-glucose (NG, 5.5 mM glucose and 20 mM mannitol) or high-glucose (HG, 25.6 mM glucose) medium for 7 days and transfected with TRPV2 siRNA or scrambled siRNA control. Values are shown as the mean ± SEM; n = 5. *P < 0.05 analyzed by two-way analysis of variance followed by Bonferroni test; ns, not significant.

4. Role of TRPV2 and reactive oxygen species in high glucose–induced mitochondrial membrane potential decrease of lens epithelial cells

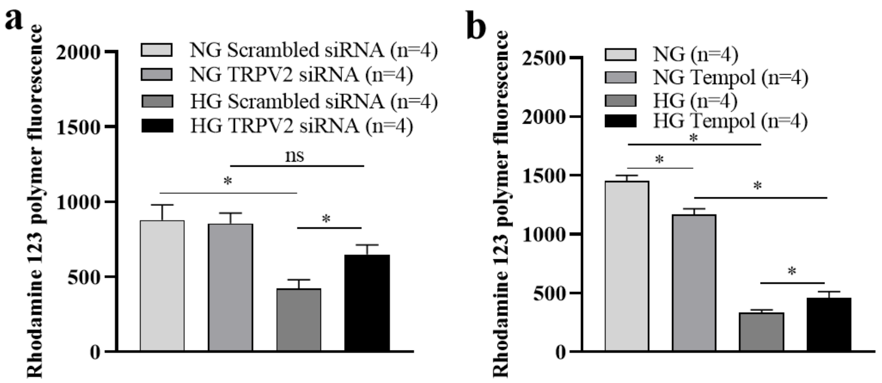

Figure S4. (**a**) Summary data showing the changes in mitochondrial membrane potential of HLEpiCs transfected with TRPV2 siRNA or scrambled siRNA control and cultured in a normal-glucose (NG, 5.5 mM glucose and 20 mM mannitol) or high-glucose (HG, 25.6 mM glucose) medium for 7 days. (**b**) Summary data showing the changes in mitochondrial membrane potential of HLEpiCs cultured in NG or HG medium with or without Tempol (1 mM, an antioxidant) treatment for 7 days. 2-way ANOVA was used to compare the significance in panels **a** and **b**. Values are shown as the mean ± SEM; n = 4. *$P < 0.05$ analyzed by two-way analysis of variance followed by Bonferroni test; ns, not significant.
